# Comparison of the regressive effects of aflibercept and brolucizumab on pigment epithelial detachment

**DOI:** 10.1186/s12886-022-02617-2

**Published:** 2022-09-29

**Authors:** Ryo Mukai, Hidetaka Matsumoto, Kazuki Nagai, Hideo Akiyama

**Affiliations:** Department of Ophthalmology, Gunma University Graduate School of Medicine, 3-35-15 Showa-cho, Maebashi, Gunma 371-8511 Japan

**Keywords:** Aflibercept, Age-related macular degeneration, Brolucizumab, Pigment epithelial detachment

## Abstract

**Background:**

To compare the regressive effects of aflibercept and brolucizumab on pigment epithelial detachment (PED) in age-related macular degeneration.

**Methods:**

Eighty-three eyes of 83 patients diagnosed with type 1 macular neovascularization were included and retrospectively analysed using multimodal imaging. Forty-nine eyes were treated with intravitreal aflibercept injections (IVA group), and 34 eyes were treated with brolucizumab (IVBr group), with three consecutive injections administered as induction therapy. Before treatment and 1, 2, and 3 months after the first treatment, the maximum height (MH) and maximum diameter (MD) of the PED were measured using optical coherence tomography in each treatment group.

**Results:**

In the IVA group, MH at baseline (228 ± 169 μm) diminished to 180 ± 150 (*P* = 0.2558), 165 ± 140 (*P* = 0.0962), and 150 ± 129 µm (*P* = 0.0284) at 1, 2, and 3 months after treatment, respectively; the reduction at 3 months was significant. In contrast, in the IVBr group, the MH was 307 ± 254 µm before treatment, and it decreased to 183 ± 156 µm (*P* = 0.0113), 139 ± 114 µm (*P* = 0.0003), and 125 ± 126 µm (*P* < 0.0001) at 1, 2, and 3 months after treatment, respectively, and the reduction at 1 month was significant. In both groups, the MD did not regress significantly.

**Conclusions:**

The results suggested that the MH of PED after IVBr treatment regressed faster than that after IVA treatment.

**Supplementary Information:**

The online version contains supplementary material available at 10.1186/s12886-022-02617-2.

## Background

Age-related macular degeneration (AMD) is a significant cause of blindness worldwide. Since 2000, anti-vascular endothelial growth factor (VEGF) drugs have been used to treat exudative lesions of AMD. To date, formulations of bevacizumab [1], pegaptanib [2, 3], ranibizumab [4], and aflibercept [5] have been used to stabilize the disease and thus improve vision. Intensive research has also yielded more potent and longer-acting drugs to treat this disease. One such drug, brolucizumab [6, 7], was launched in the United States in 2020 and is now available worldwide. Pigment epithelial detachment (PED) is closely associated with neovascular AMD. Exudative change in the retina with shallow PED indicates the presence of macular neovascularization, especially in the elderly [8]. The presence of a PED which develops due to macular neovascularization (MNV) can cause subretinal fluid, intraretinal fluid, subretinal pigmental epithelial fluid and subretinal or subretinal pigment epithelial (sub-RPE) haemorrhage, with loss of visual acuity [9]. In addition, a large PED associated with MNV can lead to the emergence of RPE tear [10]. Brolucizumab has a strong effect on subretinal pigment epithelial choroidal neovascularization or sub-RPE fluid. HAWK and HARRIER studies revealed that the percentage of patients with sub-RPE fluid treated with intravitreal brolucizumab injections (IVBr) was significantly lower than that of patients treated with intravitreal aflibercept injections (IVA) [11]. Therefore, in this study, we focused on the regressive effect of brolucizumab on PED, and compared the effects of IVA and IVBr in a real-world setting.

## Methods

Institutional review board approval for this retrospective study was obtained from Gunma University Graduate School of Medicine, and the study adhered to the Declaration of Helsinki. All patients with a clinical diagnosis of type 1 MNV and previously untreated neovascular AMD (nAMD) at the Department of Ophthalmology of Gunma University Medical Hospital between June 2015 and January 2021 were included in this study. All participants were examined using fundus ophthalmoscopy, fluorescein angiography (FA), IA (Heidelberg Engineering, Heidelberg, Germany), and swept-source optical coherence tomography (OCT; DRI OCT Triton; Topcon, Tokyo, Japan). The DRI OCT triton incorporated a tuneable laser with a central wavelength of 1050 nm and acquired 100,000 A-scans/s. SS-OCT had an axial resolution of 2.6 μm and a lateral resolution of 20 μm. SS-OCT volume images were obtained using a radial scan protocol, which covered an area of 9 × 9 mm centred on the fovea. In addition, 12-mm horizontal and vertical scans at the fovea that contained 1024 A-scans were obtained and analysed. To evaluate PED regression before and at 1, 2, and 3 months after the first treatment, the maximum height (MH) and maximum diameter (MD) of PED were measured using OCT images in each treatment group. At the initial visit and 3 months after the first treatment, best-corrected visual acuity (BCVA), central macular thickness (CMT), and central choroidal thickness (CCT) were examined. MH was defined as the distance between the RPE and Bruch’s membrane. MD was assessed by measuring the maximum expansion of the PED using radial OCT or crossed-line images. CMT was defined as the distance between the internal limiting membrane and the RPE at the fovea, and CCT was defined as the distance between Bruch’s membrane and the margin of the choroid and sclera under the fovea. MH, MD, CMT, and CCT were measured using a computer-based calliper and were recorded independently by two examiners blinded to patient information. The diagnostic criteria for nAMD were based on a previous study. Three monthly injections of brolucizumab (Beovu; 6.0 mg/0.05 mL; Novartis) or aflibercept (Eylea; 20 mg/0.05 mL; Bayer) were administered as a loading-phase treatment, depending on when the participants had visited the hospital: IVA was administered in 2015 and IVBr was administered in 2021. The emergence of idiopathic orbital inflammation (IOI) was monitored monthly. To detect vascular changes in detail, an ultra-wide field scanning laser ophthalmoscope (Optos 200Tx) was used during monitoring. In addition, we assessed retinal haemorrhage and retinal pigment epithelial tears using OCT images, fundus photographs, and autofluorescein images.

### Statistics

The Mann–Whitney *U* test was used to compare the mean age between the IVA and IVBr groups. One-way ANOVA was used to assess changes in MH and MD in each group. The Fisher test was performed to analyse the odds ratio and *p*-value for the dominance of male or female patients and prevalence of IOI, retinal haemorrhage, and RPE tear in each group. Data analysis was performed using GraphPad Prism version 9 (GraphPad Software, La Jolla, CA, USA). Statistical significance was set at *P* < 0.05. All data are presented as mean ± standard deviation.

## Results

**Fig. 1:**
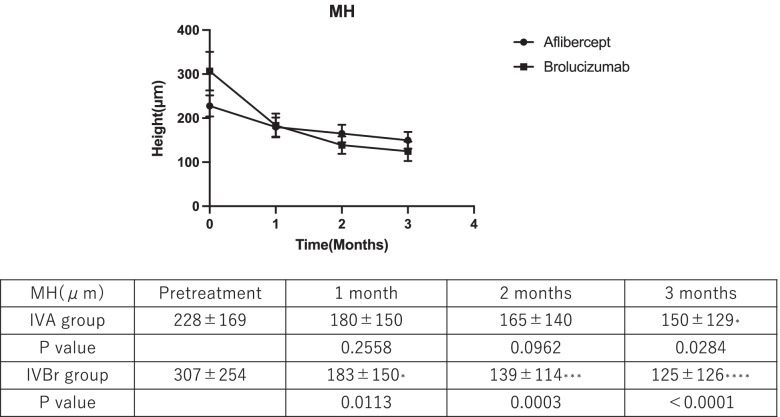
Changes in the maximum height (MH) of pigment epithelial detachment measured in the intravitreal aflibercept (IVA) and intravitreal brolucizumab (IVBr) groups before and at 1, 2, and 3 months after the first treatment. *: *P* < 0.05, ***: *P* < 0.001, ****: *P* < 0.0001

**Fig. 2:**
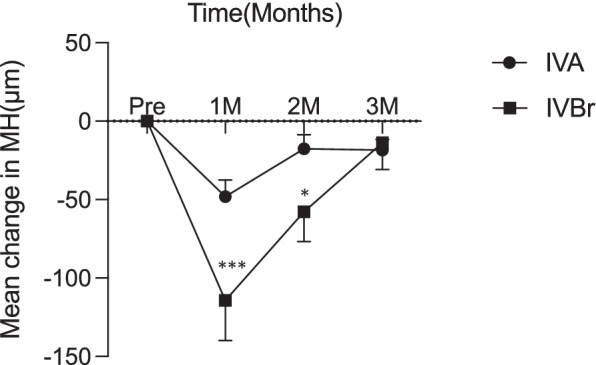
Mean changes from baseline in the maximum height (MH) of pigment epithelial detachment measured in the intravitreal aflibercept (IVA) and intravitreal brolucizumab (IVBr) groups before and at 1, 2, and 3 months after the first treatment. *: *P* < 0.05, ***: *P* < 0.001

The demographic characteristics of the IVA and IVBr groups are summarized in Table 1. The two groups showed no significant differences in age or sex distribution. There was no significant difference in mean MHs for each group at baseline (*P* = 0.11). The mean MHs of the PED before and at 1, 2, and 3 months after the first treatment in the IVBr group were 307 ± 254, 183 ± 150, 139 ± 114, and 125 ± 126 μm, respectively. The MH showed significant regression 1 month after the first treatment when compared to the baseline. In contrast, the MHs in the IVA group before and at 1, 2, and 3 months after the first treatment were 228 ± 169, 183 ± 150, 165 ± 140, and 150 ± 129 μm, respectively, and the MH significantly decreased only at 3 months after the first treatment (Fig. 1). At 1 and 2 months, the mean decreases of the MH in the IVBr were -114.3 and -57.71 μm and those in the IVA were -48.12 and -17.51 μm respectively, with statistically significant differences at the periods (*P* = 0.0088 and 0.0391, Fig. 2).

**Table 1:** Demographic characteristics in the intravitreal aflibercept (IVA) and intravitreal brolucizumab (IVBr) groups

	IVA group	IVBr group	*P* value
Eyes	49	34	
Mean age	74 ± 9	76 ± 9	0.4101
Male/Female	36/13	28/6	0.4303

**Fig. 3:**
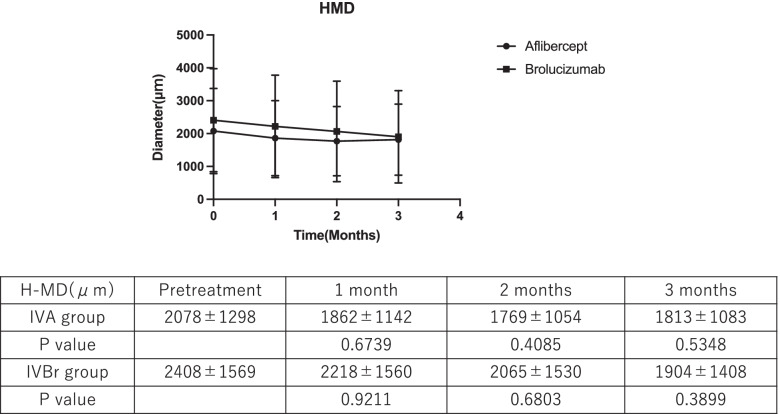
Changes in the horizontal maximum diameter (H-MD) of pigment epithelial detachment in the intravitreal aflibercept (IVA) and intravitreal brolucizumab (IVBr) groups before and at 1, 2, and 3 months after the first treatment. *; *P* < 0.05

The MDs before and at 1, 2, and 3 months after the first treatment in the IVBr group were 2408 ± 1569, 2218 ± 1560, 2065 ± 1530, and 1904 ± 1408 μm, respectively, and 2078 ± 1298, 1862 ± 1142, 1769 ± 1054, and 1813 ± 1083 μm in the IVA group, respectively. In both groups, MD before and after the treatment did not change significantly (Fig. 3).

The prevalence of worsened retinal haemorrhage was 4/49 in the IVA group and 1/34 in the IVBr group, with no significant difference in the incidence between the two groups (*P* = 0.6440). Additionally, the incidence of RPE tears was 5/49 in the IVA group and 1/34 in the IVBr group, with no significant difference between groups (*P* = 0.3930). Moreover, IOI occurred in 5/34 patients in the IVBr group but not in the IVA group (Table 2). In all cases, local triamcinolone was administered, the IOI subsided, and visual function was mostly restored.

**Table 2:** Prevalence of retinal pigment epithelial tear, worsening of retinal haemorrhage, and intraocular inflammation in the intravitreal aflibercept (IVA) and intravitreal brolucizumab (IVBr) groups at 3 months after the first treatment

	IVA group	IVBr group	*P* value
Eyes	49	34	
RPE tear	5(10%)	1(3%)	0.3930
Worsening of retinal hemorrhage	4(8%)	1(3%)	0.6440
Intra ocular inflammation	0	5(15%)	0.0096 (95%CI:0–0.5096)

*RPE* Retinal pigment epitheial

**Fig. 4:**
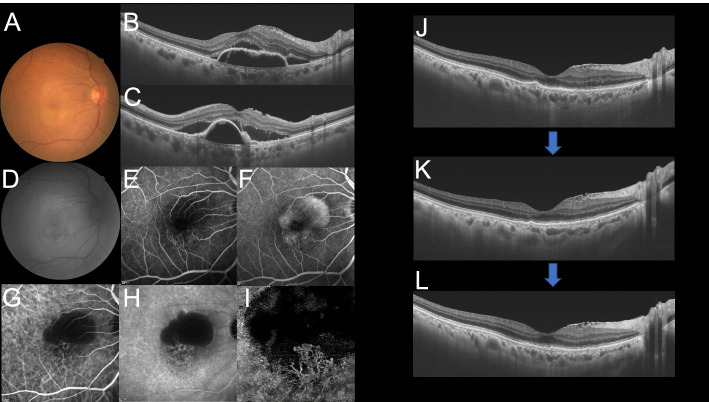
A 75-year-old male in the intravitreal brolucizumab (IVBr) treatment group at base line (**A**-**I**). **A** Fundus photograph showed pigment epithelial detachment (PED) at the macula. **B**, **C** Optical coherence tomography (OCT; horizontal (**B**) and vertical images (**C**) revealed PED with subretinal fluid. **D** Fundus autofluorescence image. **E**, **F** Early and late phase of fluorescein angiography detected occult macular neovascularization (MNV) at the macula. **G**, **H** Early and late phase of indocyanine green angiography identified MNV at the bottom of PED **I** OCT angiography showed MNV at the bottom of the lesion. OCT images of the case at 1,2 and 3 months after IVBr treatment (**J**-**L**). At 1 month after treatment, PED dramatically regressed (**J**, **K**). At 2 months, the PED gradually reduced and almost disappeared at 3 months (**L**)

In addition, the prevalence of macular dryness in the IVA and IVBr groups was 42/49 (86%) and 30/34 (88%), respectively (*P* = 0.99). Changes in CMT, CCT, and BCVA are summarized in Additional file 1. The representative case treated with IVBr is shown in Fig. 4.

## Discussion

Overall, IVBr treatment for type 1 MNV can achieve faster regression of PED than IVA treatment. However, the rate of occurrence of RPE tears and retinal haemorrhage did not differ between the two groups in this cohort. PED in AMD occurs due to the high vascular permeability of the MNV or physical (direct) invasion of MNV from the choroid into the space between Bruch’s membrane and RPE, which can cause bump formation in the RPE [12, 13]. As a result, serous PED or vascularized PED can form the surrounding MNV [14]. In fact, all type 1 cases in this study showed PED: 16 cases showed mostly serous PED, 31 cases showed fibrovascular PED, and 2 cases showed both fibrovascular and serous PED in the IVA group; 5 cases showed mostly serous PED and 29 cases showed fibrovascular PED in the IVBr group. Moreover, no significant difference was observed in the distribution of PED type (*P* = 0.07). Both treatments caused PED regression, possibly because of both suppression of leakage from MNV and reduction of MNV size subsequent to inhibition of vascularized PED formation by MNV.

Marco R and his colleagues investigated the morphological changes in PED treated with a single brolucizumab shot in refractory cases in comparison with the effects after previous ranibizumab or aflibercept treatment. In their report, brolucizumab could diminish PED size in accordance with CNV regression in such cases [15]. For polypoidal choroidal vasculopathy, a previous study revealed that IVBr can achieve a higher polyp occlusion rate than IVA treatment. We reported 78.9% complete occlusion of polyps after IVBr treatment following induction therapy [16]. Similar findings were published by Fukuda and his colleagues [17]. In contrast, IVA monotherapy could occlude polyps at the rate of 55% [18]. Although these studies evaluated polyp occlusion using indocyanine green angiography, their results strongly suggested that IVBr can regress PED more strongly than IVA can.

In this study, we tried an additional comparison of MH of PED; briefly, we divided the participants into two groups, a group with ≥ 300 μm height of PED and a group with < 300 μm in each treatment group. Then we analysed the change in the MH. Significant decrease in a group with ≥ 300 μm height of PED (*n* = 24) was detected in the IVBr group (*n* = 13) at 1 month after the first treatment; contrarily, no significant changes were seen at 1 and 2 months after the treatment in the IVA group (*n* = 11). Additional Fig. 2 shows the results of this analysis.

The loading dose of IVBr caused choroidal thinning with a 16% reduction in the original thickness, which has been reported in at least two studies [16, 19]. On the other hand, while the same dosage of IVA can cause choroidal thinning, the extent was similar or lower to that of IVBr [20]. These results also support the possibility that IVBr strongly affects choroidal lesions.

In this study, RPE tears developed in 1/34 (3%) eyes treated with IVBr; in contrast, 5/49 (10%) eyes were treated with IVA. The two treatment groups showed no significant difference in the development of RPE tears (*P* = 0.3930); however, the rate in the IVBr group was lower than that in the IVA group, even though relatively large PED were present in the IVBr group. This was surprising, since it is generally feared that faster regression of the PED can lead to higher risk of RPE tear formation. It is thought that RPE tears develop in accordance with NV regression beneath the RPE, and that they can develop more frequently in cases with more elevated PEDs [10]. We now speculate that the RPE tear could be explained by the impulse experienced by the PED, which is the result of the force of contraction of the MNV multiplied by time. Since the time of PED flattening is shorter in IVBr cases, this may mean a lower overall impulse. Of course, other biological variables, including among other factors, the effect of different agents on the RPE tight junctions could also be important.

Incidental emergence of IOI was a serious issue during usage of IVBr [21, 22], which could limit the adoption of IVBr in AMD [11]. The most feared complication relates to rare but visually devastating cases of occlusive retinal vasculitis. We did not observe any cases of occlusive vasculitis in our study. We did observe IOI in 5 of our patients, but the visual function improved in two cases, was preserved in two cases, and slightly worsened in one case (20/32 to 20/40). Both early detection and immediate treatment for IOI was required during the use of IVBr.

## Conclusions

In summary, in comparison with IVA, IVBr can potentially contribute to the stability of sub-RPE lesions.

## Supplementary Information

**Additional file 1:** **Supplemantary figure 1.** Changes in central macular thickness (CMT), central choroidal thickness (CCT), and best corrected visual acuity (BCVA) at the initial visit and 3 months after the first treatment.

**Additional file 2:** **Supplementary figure 2.** Mean changes from baseline in the maximum height (MH) of pigment epithelial detachment (PED) measured in the intravitreal aflibercept (IVA) and intravitreal brolucizumab (IVBr) groups in cases with ≥ 300 μm of PED before and at 1, 2, and 3 months after the first treatment.

## Data Availability

The datasets used and/or analysed during the current study available from the corresponding author on reasonable request.

## Abbreviations

PED: Pigment epithelial detachment
IVA: Intravitreal aflibercept injections
IVBr: Intravitreal brolucizumab injections
MH: Maximum height
MD: Maximum diameter
AMD: Age related macular degeneration
MNV: Macular neovascularization
RPE: Retinal pigment epithelium
BCVA: Best-corrected visual acuity
CMT: Central macular thickness
CCT: Central choroidal thickness
OCT: Optical coherence tomography
IOI: Intra ocular inflammation
