# Supplementary material for: Comparison of the regressive effects of aflibercept and brolucizumab on pigment epithelial detachment
Source: BMC Ophthalmol. 2022 Sep 29;22:387. doi: 10.1186/s12886-022-02617-2 (PMC9520796; doi:10.1186/s12886-022-02617-2)

Supplemantary figure 1.

Changes in central macular thickness (CMT), central choroidal thickness (CCT), and best corrected visual acuity (BCVA) at the initial visit and 3 months after the first treatment

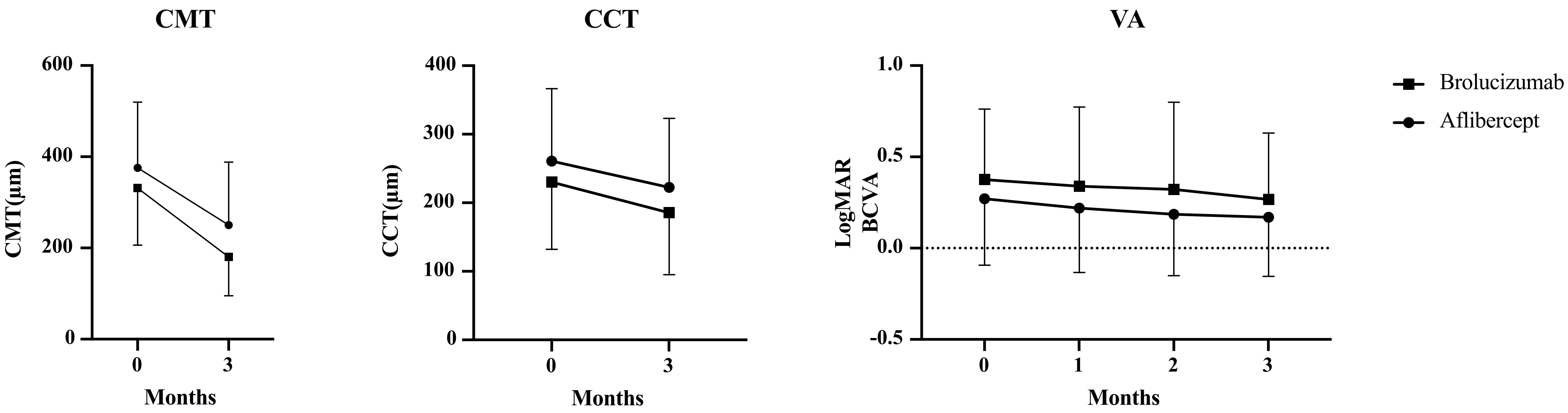

Supplement: Supplementary file 1 — Additional file 1: Supplemantary figure 1. Changes in central macular thickness (CMT), central choroidal thickness (CCT), and best corrected visual acuity (BCVA) at the initial visit and 3 months after the first treatment. [file 12886_2022_2617_MOESM1_ESM.pdf]
