# Supplementary material for: Comparison of the regressive effects of aflibercept and brolucizumab on pigment epithelial detachment
Source: BMC Ophthalmol. 2022 Sep 29;22:387. doi: 10.1186/s12886-022-02617-2 (PMC9520796; doi:10.1186/s12886-022-02617-2)

Supplementary figure 2

Mean changes from baseline in the maximum height (MH) of pigment epithelial detachment (PED) measured in the intravitreal aflibercept (IVA) and intravitreal brolucizumab (IVBr) groups in cases with ≥ 300 µm of PED before and at 1, 2, and 3 months after the first treatment.

*: P < 0.05, ***: P < 0.001, ****: P < 0.0001

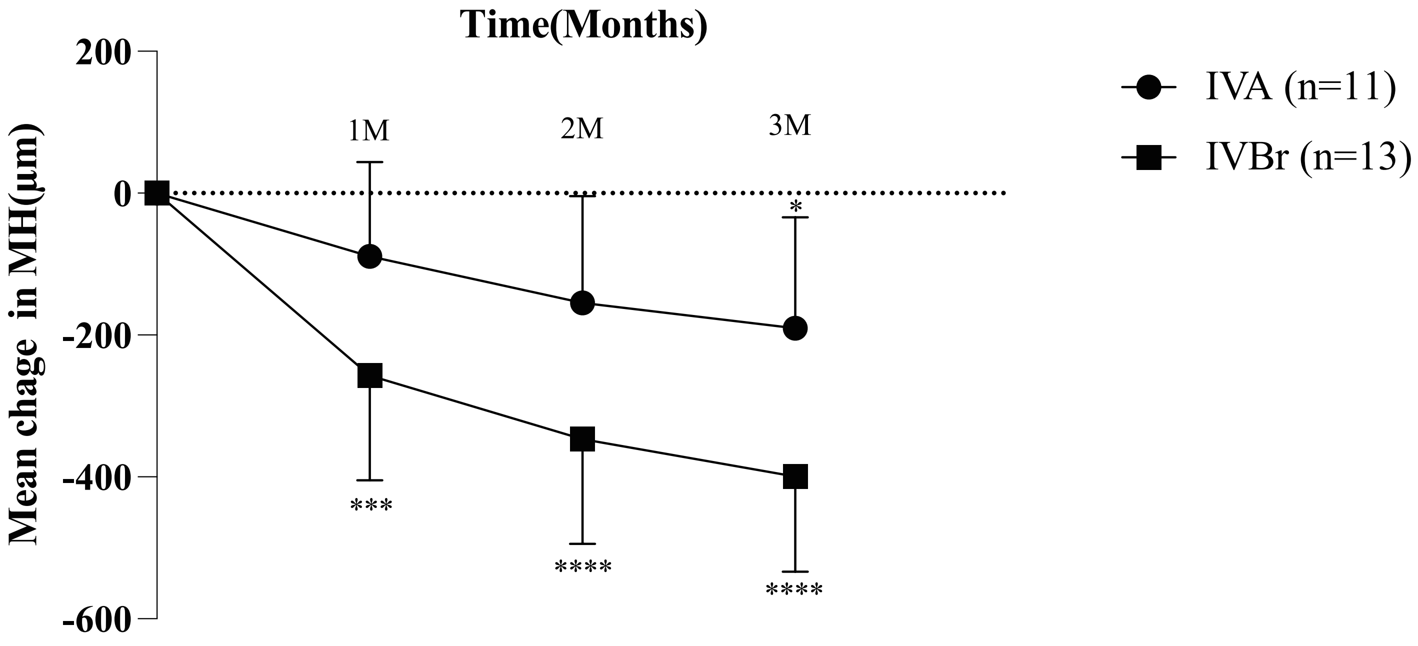

Supplement: Supplementary file 2 — Additional file 2: Supplementary figure 2. Mean changes from baseline in the maximum height (MH) of pigment epithelial detachment (PED) measured in the intravitreal aflibercept (IVA) and intravitreal brolucizumab (IVBr) groups in cases with ≥ 300 μm of PED before and at 1, 2, and 3 months after the first treatment. [file 12886_2022_2617_MOESM2_ESM.pdf]
